# Bifunctional TiO_2_@AgNP Superstructures as a SERS-Sensing Platform for Identifying Flavonoids in Chinese Herbal Medicine

**DOI:** 10.3390/bios15080536

**Published:** 2025-08-15

**Authors:** Yulin Li, Junbo Li, Haisu Wang, Shaorui Qi, Zhehao Zhang, Yaqiu Wang, Ying Wang, Wei Ji

**Affiliations:** 1 College of Chemistry, Chemical Engineering and Resource Utilization, Northeast Forestry University, Harbin 150040, China; liyulin@nefu.edu.cn (Y.L.); wangying@nefu.edu.cn (Y.W.); 2 School of Chemical Engineering, Dalian University of Technology, Dalian 116024, China

**Keywords:** surface-enhanced Raman scattering (SERS), TiO_2_@AgNPs, flavonoids, *Acanthopanax senticosus*

## Abstract

*Acanthopanax senticosus* is an essential medicinal herb in traditional Chinese medicine, with its pharmacological properties largely attributed to bioactive flavonoids. The types and amounts of these flavonoids act as vital quality markers for both the raw materials and the resultant products. In this work, we introduce a TiO_2_@AgNP nanocomposite designed as a surface-enhanced Raman scattering (SERS) sensor aimed at the preliminary quantification and identification of flavonoids. This is achieved by leveraging the effective molecular adsorption properties of TiO_2_ alongside the ‘hot spots’ generated by AgNPs. By optimizing SERS performance through adjustment of the molar ratio between TiO_2_ and Ag, we can quantitatively evaluate four flavonoids—luteolin, kaempferol, quercetin, and rutin—with low detection concentrations of 10^−6^ M, 10^−5^ M, 5 × 10^−6^ M, and 10^−6^ M, respectively. Additionally, we observe a nearly linear relationship between the SERS signals and the flavonoid concentrations, allowing for dual or multiplex analysis of these compounds. Furthermore, we successfully differentiated *Acanthopanax senticosus* samples from six different geographical regions in China based on the detection of significant flavonoid constituents. This serves as a proof of concept for practical applications that can enhance the identification and distinction of traditional Chinese medicine, as well as assess quality and medicinal efficacy.

## 1. Introduction

*Acanthopanax senticosus* (*AS*), widely recognized as Siberian Ginseng in the UK, Ciwujia in China, and Shigoka in Japan, is a perennial herbaceous plant belonging to the Araliaceae family. This medicinal species is mainly found in China, Japan, and South Korea, particularly in the northeastern provinces of Heilongjiang, Jilin, and Liaoning in China [1]. The different parts of *AS*, such as the root, bark, leaves, and fruit, demonstrate a variety of pharmacological effects, including antioxidant and anti-inflammatory properties, along with potential uses in treating neurodegenerative diseases [2,3,4,5]. Furthermore, *AS* is widely accepted due to its mild therapeutic properties, low toxicity levels, and ability to target multiple pathways, which has led to its prevalent inclusion in health foods and traditional Chinese medicine [6,7]. Nevertheless, challenges related to quality control continue to arise due to unregulated factors in growth conditions, harvesting techniques, and processing methods. Moreover, problems like adulteration, contamination, and elevated levels of heavy metals pose additional risks, resulting in variable therapeutic effects across different batches of *AS*. Hence, it is essential to improve quality control measures, which can be accomplished through enhanced monitoring and quantitative analysis of active constituents. Among the bioactive constituents of *AS*, flavonoids are acknowledged as crucial components, displaying pharmacological activities such as antioxidant, antitumor, and vasoprotective effects [8,9,10,11]. The quantity of flavonoids is an important criterion for assessing the quality of *AS*. Currently, various analytical methods, mainly chromatography and mass spectrometry, are utilized for detecting flavonoids [12,13,14]. However, practical challenges, including operational difficulties, specialized requirements, and high equipment costs, limit their use for prompt, high-throughput, and ultrasensitive flavonoid detection in actual samples [15]. This situation necessitates the development of alternative analytical tools to overcome these limitations.

Surface-enhanced Raman spectroscopy (SERS) has emerged as a powerful analytical technique, offering numerous advantages, including exceptional sensitivity, non-destructive analysis, and the capacity to provide unique molecular fingerprints [16,17,18]. These features facilitate the direct and rapid detection of analytes such as pesticides, pollutants, toxic compounds, and biomolecules across diverse fields, such as environmental monitoring, food safety, and biomedical research [19,20,21,22,23,24]. Commonly utilized SERS substrates often consist of nanostructured metallic solids or nanoparticle solutions, which create a significant electromagnetic enhancement field that plays a crucial role in amplifying the SERS signal. Specifically, silver or gold colloids have been tailored for examining traditional Chinese medicine. For instance, silver nanospheres or those modified with cesium ions have been employed to analyze the chemical compositions and quality of samples from different areas [25,26,27,28]. An interface-based plasmonic gold nanoarray has been introduced as a SERS-sensing platform aimed at the initial quantification and identification of active constituents, including flavonoids [29]. Moreover, semiconductor nanoparticles have emerged as effective substrates for SERS due to their unique features, with titanium dioxide (TiO_2_) being one of the most extensively investigated candidates because of its good spectral uniformity, biocompatibility, and superior oxidation resistance [30,31,32,33]. Importantly, flavonoids containing hydroxyl groups facilitate effective adhesion to the TiO_2_ surface through chemical interactions between these hydroxyl functionalities (–OH) and titanium atoms at the surface (Ti^4+^ sites). This interaction reduces the distance between the analyte and TiO_2_, significantly enhancing its capability as a substrate for the detection of flavonoids in *AS*. Nonetheless, the relatively low enhancement factor associated with semiconductor-based SERS substrates limits their further utilization in flavonoid analysis [34]. To address this issue, hybrid composites that integrate noble-metal nanoparticles with semiconductor nanoparticles present a promising approach for increasing SERS signal intensities, leveraging the combined effects of electromagnetic enhancement and a larger surface area that enables a greater quantity of adsorbed molecules.

**Figure 1:**
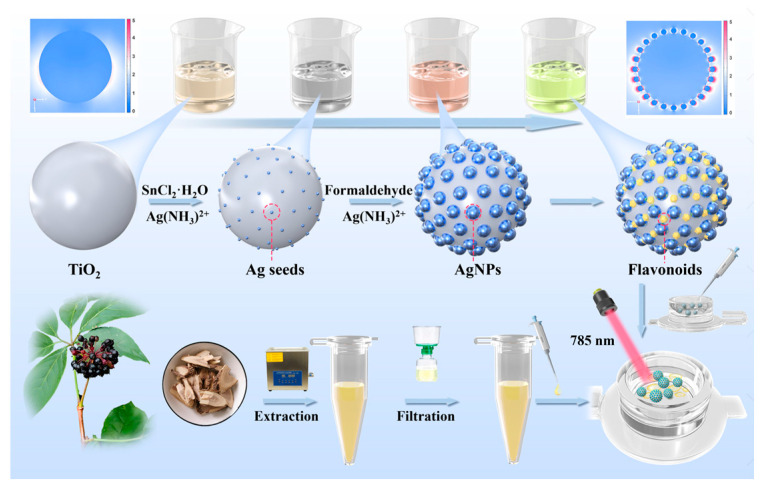
Schematic illustration of the preparation of TiO_2_@AgNPs, which serve as a SERS sensor for the analysis of flavonoids in *Acanthopanax senticosus*.

Motivated by these findings, we developed a nanocomposite of noble metals and semiconductors, referred to as TiO_2_@AgNPs, intended to act as a SERS sensor for flavonoid analysis (Figure 1). The synthesis of this sensor was achieved by chemically reducing silver nanoparticles on the surface of amorphous-phase TiO_2_, which features active sites. By varying the molar ratio of TiO_2_ to Ag, we pinpointed the composition of TiO_2_@AgNPs that displayed superior SERS performance. Importantly, the TiO_2_@AgNPs successfully detected four flavonoids—luteolin, quercetin, kaempferol, and rutin—which correspond to flavones, flavonols, and flavonoid glycosides, respectively, demonstrating excellent signal repeatability. A notable linear correlation was found between the SERS signals and the concentrations of the standard flavonoids. Additionally, the detection of dual or multiplex flavonoids was accomplished, indicating potential for applications in analyzing practical samples. To validate this concept, SERS evaluations were performed on samples of *AS* sourced from six different geographical regions in China, demonstrating that TiO_2_@AgNPs have a commendable ability to discriminate based on the primary flavonoid constituents in *AS*. Taken together, this SERS sensor provides a straightforward approach for assessing both the qualification and quantification of bioactive components in *AS*, thereby underscoring its substantial potential for identifying and distinguishing features in traditional Chinese medicine and further aiding in quality assessment.

## 2. Materials and Methods

### 2.1. Materials and Reagents

Silver nitrate was obtained from Sigma-Aldrich. Tetrabutyl titanate, thioglycolic acid, ethanol, acetic acid, luteolin, rutin, quercetin, kaempferol, formaldehyde, and ammonia solutions were purchased from Aladdin China Co., Ltd (Shanghai, China). *Acanthopanax senticosus* (*AS*), sourced from Shanxi, Hubei, Heilongjiang, Sichuan, Liaoning, and Jilin, was acquired via the online platform at https://www.zyccst.com/ (accessed on 12 August 2025). All chemicals used were of analytical grade and were used as received without further purification. Milli-Q water was utilized for the preparation of all solutions used in this research.

### 2.2. Characterization Methods

TEM images were acquired using a JEM-2100 microscope (JEOL Ltd., Tokyo, Japan) operating at an accelerating voltage of 200 kV. SEM images were obtained with a JSM-7500F field emission scanning electron microscope (JEOL Ltd., Tokyo, Japan). XRD spectra were collected using a TD-3500X-ray diffractometer (Dandong Tongda Technology Co., Ltd., Dandong, China) in reflection mode with Cu Kα radiation (30 kV, 20 mA). Measurements of Raman and SERS spectra were executed with a Zolix laser confocal Raman spectrometer set at an excitation wavelength of 785 nm. All analyses were conducted at ambient temperature.

### 2.3. Synthesis of TiO_2_ Nanoparticles

Amorphous-phase TiO_2_ nanoparticles were synthesized using the sol–gel method [35]. Specifically, 1 mL of tetrabutyl titanate and 6 μL of thioglycolic acid were combined with 14 mL of ethanol, maintaining continuous stirring for 3 h. Subsequently, 1 mL of deionized water was introduced into the reaction mixture, which was stirred for an additional 3 h. Finally, the reaction mixture was subjected to centrifugation, and the resulting product was washed several times with ethanol to yield TiO_2_ nanoparticles with an amorphous structure.

### 2.4. Synthesis of TiO_2_@AgNP Nanocomposites

TiO_2_@AgNP nanocomposites were synthesized by depositing AgNPs onto TiO_2_ nanoparticles [36]. Initially, 0.02 g of TiO_2_ nanoparticles was dispersed in 20 mL of deionized water containing 0.014 g of SnCl_2_·H_2_O. The mixture was stirred vigorously at room temperature for 30 min to activate the TiO_2_ nanoparticles. Subsequently, the activated TiO_2_ nanoparticles were introduced into Ag(NH_3_)^2+^ solutions of varying concentrations and stirred thoroughly for an additional 30 min. The resultant mixture underwent centrifugation, filtration, and extensive washing with deionized water to isolate the TiO_2_ nanoparticles embedded with Ag seeds. Following this, the TiO_2_ nanoparticles containing Ag seeds were reintroduced into the Ag(NH_3_)^2+^ solution and stirred. Meanwhile, a mixed solution of formaldehyde and ethanol was prepared by combining 0.4 mL of formaldehyde solution, 0.4 mL of deionized water, and 9.2 mL of ethanol. Subsequently, 2 mL of this mixed solution was added dropwise to the suspension of TiO_2_ nanoparticles containing Ag seeds under continuous stirring. The resulting mixture was stirred for an additional 30 min. Finally, the product was subjected to centrifugation, filtration, washing, and vacuum drying at 80 °C for 12 h to obtain TiO_2_@AgNPs.

### 2.5. Preparation of AS Extracts

*AS* extracts were pulverized using a grinder and subsequently sieved through a 40-mesh sieve prior to analysis. A total of 1.5 g of the powdered sample from *AS* roots was mixed with 20 mL of methanol and subjected to ultrasonic extraction at a frequency of 100 kHz and a temperature of 40 °C for 1 h. The resulting solution was filtered through a vacuum filtration system to remove coarse residues, followed by a final filtration using a 0.45 μm microporous membrane.

### 2.6. SERS Measurement Protocol

Initially, an aluminum foil strip was adhered to the inner surface of a 2 mL centrifuge tube cap. Subsequently, 6 μL of a 1 mg/mL TiO_2_@AgNP nanocomposite suspension was deposited onto the foil and allowed to evaporate, facilitating the formation of an active substrate. Following this, 100 μL of the analyte solutions, including flavonoid standards and *AS* extracts, was applied to the substrate, air-dried, and reapplied for two additional cycles to enhance analyte adsorption. During Raman spectroscopy testing, all wavelengths were maintained within the range of 300 to 1800 cm^−1^. All SERS measurements were performed directly using a spectrometer equipped with a 785 nm laser. The SERS spectra were recorded under the following parameters: a laser power of 16.5 mW, an acquisition time of 6 s, and 1 scan cycle. The acquired SERS spectra were processed via baseline correction, signal smoothing, and normalization prior to further analysis.

## 3. Results

### 3.1. Optimization and Structural Characterization of TiO_2_@AgNPs

**Figure 2:**
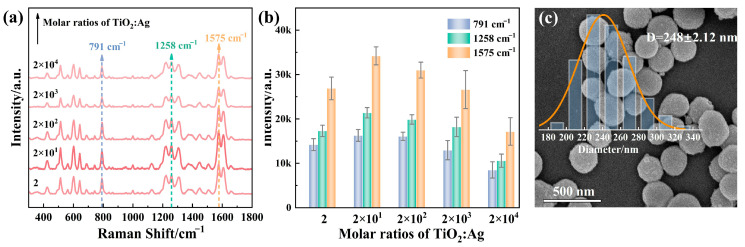
(**a**) SERS spectra for luteolin (10^−4^ M) on TiO_2_@AgNPs synthesized using varying molar ratios of TiO_2_ to Ag, ranging from 2 to 2 × 10^4^. (**b**) SERS intensities of the luteolin peak at 791 cm^−1^, 1258 cm^−1^, and 1575 cm^−1^ corresponding to the molar ratios of TiO_2_ to Ag. (**c**) SEM images of TiO_2_@AgNPs prepared with a molar ratio of TiO_2_ to Ag of 2 × 10^1^.

The deposition density of AgNPs on TiO_2_ nanoparticles significantly influences the abundance of SERS ‘hot spots’, which are crucial for electromagnetic enhancement in SERS performance. At low densities, the considerable gaps between spatially isolated AgNPs not only fail to generate the strong electromagnetic fields necessary for effective ‘hot spots’ but also hinder the formation of Ti-O-Ag interfacial bonding sites. Conversely, an excessive amount of AgNPs can lead to nanoparticle aggregation, thereby attenuating resonant plasmonic coupling. Overcrowded AgNPs obscure the active sites on the TiO_2_ surface, thus impairing flavonoid adsorption and reducing the sensitivity of analyte detection. To optimize the amount of AgNPs, the UV-Vis absorption and SERS properties of TiO_2_@AgNPs with initial molar ratios of TiO_2_ to Ag, varying from 2 to 2 × 10^4^, were investigated. As observed from the UV-vis spectra, TiO_2_@AgNPs with a molar ratio of 2 × 10^1^ exhibited wide absorption in the range of 400–800 nm, which may be due to the plasmon coupling between adjacent nanoparticles with a suitable particle size (Figure S1). Moreover, the 785 nm wavelength minimizes fluorescence interference from the samples, making it particularly suitable for detecting complex biological samples. Therefore, we opted to use the 785 nm wavelength for all subsequent testing to enhance the quality of our results. Then, luteolin was employed as a model SERS analyte. As shown in Figure 2a, SERS measurements for luteolin at a concentration of 10^−4^ M were performed. The SERS intensity at the peaks of 791 cm^−1^, 1258 cm^−1^, and 1575 cm^−1^, which correspond to ring breathing, OH bending, and C=C stretching [37], initially increased until the molar ratio of TiO_2_ to Ag reached 2 × 10^1^ (Figure 2b). Subsequently, a slight decrease in intensity was observed with further increases in molar ratios. This trend suggests that the optimal conditions for achieving the maximum effective ‘hot spots’ and the best molecular adsorption may occur at a molar ratio of 2 × 10^1^, which is further corroborated by the diminished signals observed in Figure S2. Therefore, a molar ratio of 2 × 10^1^ was employed for subsequent experiments. Furthermore, the microstructure of TiO_2_@AgNPs at this molar ratio was analyzed using scanning electron microscopy (SEM), which demonstrated the uniformity of the particles, with a statistically averaged diameter of approximately 248 nm, as depicted in the inset image (Figure 2c). Additional SEM characterizations of pure TiO_2_ and TiO_2_@AgNPs at other molar ratios are provided in Figure S3. Following Ag deposition, the originally smooth surfaces of TiO_2_ exhibited roughening, accompanied by an increase in all particle diameters. Theoretical simulations have confirmed the existence of a highly intense electromagnetic field in the nanogap regions between TiO_2_ and AgNPs (Figure S4). This observation suggests the formation of hot spots that may enhance the SERS signal, whereas pure TiO_2_ does not demonstrate significant hot spots.

**Figure 3:**
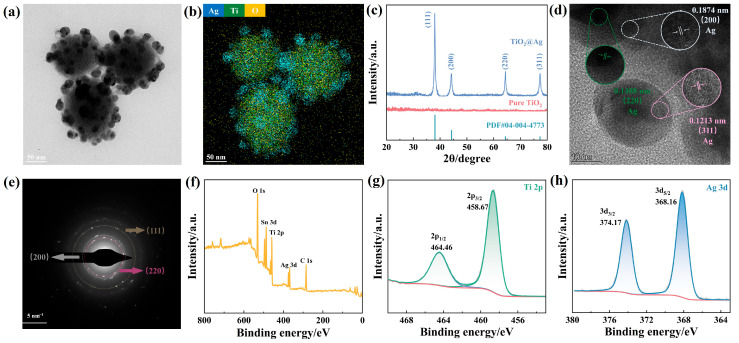
TEM (**a**), merged EDX mapping (**b**), XRD (**c**), HRTEM (**d**), and SAED (**e**) images of TiO_2_@AgNPs. Full-range (**f**), Ti 2p (**g**), and Ag 3d (**h**) XPS spectra of TiO_2_@AgNPs.

The transmission electron microscopy (TEM) image of TiO_2_@AgNPs, as illustrated in Figure 3a, provides further insights into their morphology. Discrete AgNPs, which are predominantly spherical and uniformly distributed, are observed on the surfaces of TiO_2_. Notably, despite the high loading of Ag, substantial areas of the TiO_2_ substrate remain exposed, thereby preserving accessible active sites. The elemental distributions of Ag, O, and Ti on the corresponding TiO_2_@AgNPs are analyzed using energy-dispersive spectroscopy (EDS) mapping, confirming the successful immobilization of AgNPs on TiO_2_ (Figure 3b and Figure S5). The crystalline structure of TiO_2_@AgNPs was subsequently characterized using powder X-ray diffraction (XRD). Four distinct diffraction peaks are clearly observed at 2θ values of 38.0°, 44.2°, 64.3°, and 77.3°, corresponding to the (111), (200), (220), and (311) crystalline planes of Ag (Figure 3c). It is noteworthy that the XRD pattern does not display any diffraction peaks for titanium, which may be attributed to the low crystallinity of the TiO_2_ synthesized via this method, resulting in an amorphous form. Moreover, the high-resolution transmission electron microscopy (HRTEM) image presented in Figure 3d displays three distinct crystal lattice fringes: one measuring 0.1874 nm, highlighted in white; another at 0.1488 nm, marked in green; and a third at 0.1213 nm, indicated in pink. These measurements correspond to the (200), (220), and (311) crystalline planes of Ag, respectively. Furthermore, the (111) lattice planes of Ag are noticeably absent in the HRTEM and are also missing in the selected area electron diffraction (SAED) image depicted in Figure 3e.

In order to elucidate the valence states of Ti and Ag, X-ray photoelectron spectroscopy (XPS) measurements were conducted. Figure 3f illustrates the complete XPS spectrum for TiO_2_@AgNPs, which displays notable peaks associated with Ti, Ag, Sn, O, and C, with binding energies recorded at approximately 458.77 eV (Ti 2p), 368.22 eV (Ag 3d), 487.01 eV (Sn 3d), 530.79 eV (O 1s), and 285.03 eV (C 1s). The presence of Sn is likely attributed to the incorporation of SnCl_2_∙H_2_O during the surface activation process for Ag deposition on TiO_2_. Figure 3g displays the high-resolution peaks of Ti 2p_3/2_ and Ti 2p_1/2_ from TiO_2_@AgNPs. The binding energies for Ti found at 458.67 eV and 464.46 eV in TiO_2_@AgNPs align with the previously reported Ti^4+^ states in TiO_2_ [38]. The observed peak locations and the separation between the 2p_1/2_ and 2p_3/2_ peaks, which is measured at approximately 5.79 eV, further confirm the +4 oxidation state of the Ti element, which is characteristic of TiO_2_. Notably, the Ti 2p peaks demonstrate slight energy shifts toward lower values compared to typical TiO_2_ binding energies, likely due to oxygen vacancies within the TiO_2_ lattice that facilitate electron trapping by Ti atoms. Figure 3h illustrates the XPS spectra of the Ag 3d_5/2_ and Ag 3d_3/2_ doublet peaks from the TiO_2_@AgNPs, positioned at 368.16 eV and 374.17 eV, respectively, which align closely with the values for elemental Ag [39].

### 3.2. Establishment of the SERS Sensor for Flavonoid Detection and Quantitative Analysis

**Figure 4:**
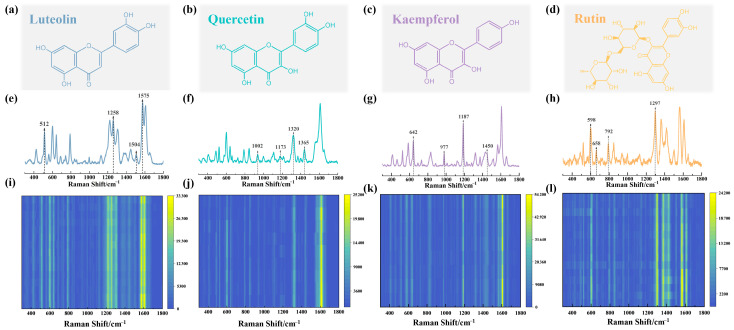
The chemical structures (**a**–**d**), typical SERS spectra (**e**–**h**), and SERS heatmaps (**i**–**l**) for luteolin (**a**,**e**), quercetin (**b**,**f**), kaempferol (**c**,**g**), and rutin (**d**,**h**), all at a concentration of 10^−3^ M.

Four flavonoids—luteolin, quercetin, kaempferol, and rutin—were selected as representatives of flavones, flavonols, and flavonoid glycosides for measurement (Figure 4a–d). Standard solutions of these flavonoids were prepared in ethanol and subsequently dropped onto the TiO_2_@AgNP substrate. The hydroxyl groups within the flavonoids promote their adsorption on the TiO_2_ surface, while the evaporation of ethanol facilitates the migration of flavonoids into the plasmonic ‘hot spots’ created by AgNPs, a process that is critical for generating the observed SERS signals. Consequently, the resulting typical SERS spectra are presented in Figure 4e–h, with detailed peak assignments listed in Tables S1–S4. For instance, the characteristic bands at 512, 1258, 1504, and 1575 cm^−1^ for luteolin in Figure 4e are attributed to in-plane bending vibrations of OH and C=C stretching, respectively. Furthermore, the corresponding SERS heatmaps in Figure 4i–l demonstrate the better signal reproducibility when detecting flavonoids utilizing the TiO_2_@AgNP substrate.

**Figure 5:**
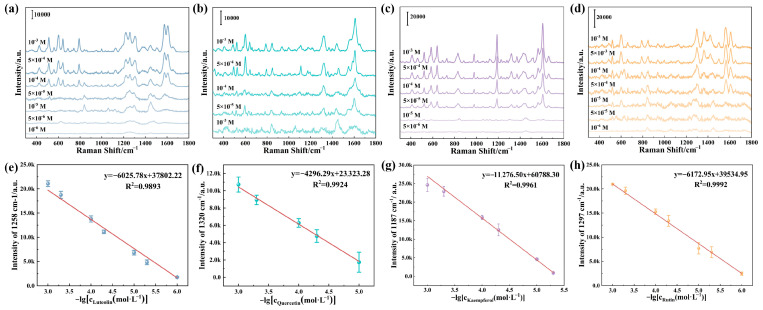
SERS spectra for luteolin (**a**), quercetin (**b**), kaempferol (**c**), and rutin (**d**) across a range of concentrations measured with TiO_2_@AgNPs. The Raman intensities at 1258 cm^−1^ for luteolin (**e**), 1320 cm^−1^ for quercetin (**f**), 1187 cm^−1^ for kaempferol (**g**), and 1297 cm^−1^ for rutin (**h**) were graphed in relation to the respective logarithmic concentrations of these flavonoids.

Subsequently, quantitative evaluations of the four flavonoids were conducted to investigate the sensitivity of TiO_2_@AgNPs. Analysis of the SERS spectra for the flavonoids at different concentrations revealed that the intensity of the characteristic peaks decreased as the concentration decreased (Figure 5a–d). The representative peaks selected for luteolin, kaempferol, quercetin, and rutin occurred at 1258, 1187, 1320, and 1297 cm^−1^, respectively, and were observable at low concentrations of 10^−6^ M, 10^−5^ M, 5 × 10^−6^ M, and 10^−6^ M. Specifically, the peak at 1258 cm^−1^ for luteolin and the peak at 1320 cm^−1^ for quercetin were attributed to in-plane bending of OH, while the peak at 1187 cm^−1^ for kaempferol was linked to the COH and C-H in-plane bending vibration, and the peak at 1297 cm^−1^ for rutin was derived from CCH bending (Tables S1–S4). Furthermore, the SERS intensities at the four peaks were plotted against the concentrations of the corresponding flavonoids, resulting in correlation curves presented in Figure 5e–h. The curves for luteolin, kaempferol, quercetin, and rutin exhibited a strong linear correlation between SERS intensity and concentration, featuring high R^2^ values of 0.9893, 0.9824, 0.9961, and 0.9992, respectively. These results demonstrate the successful SERS quantification of flavonoids using TiO_2_@AgNPs, underscoring their potential application in the practical analysis of *AS* extracts.

### 3.3. Practical Feasibility of Rapid Multiplex Analysis of Flavonoids

**Figure 6:**
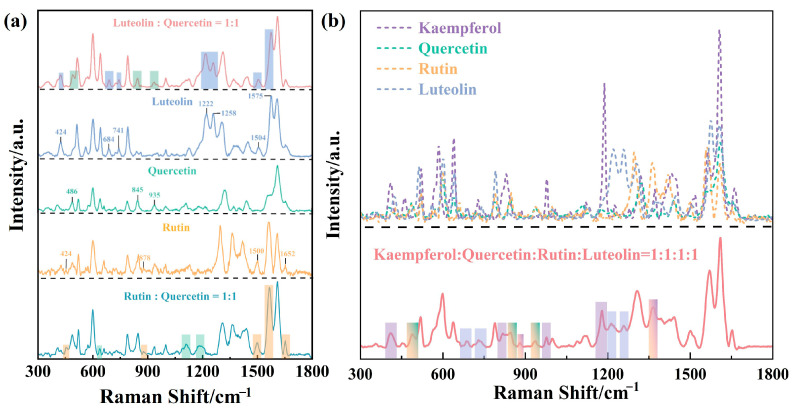
(**a**) The SERS spectra illustrate the dual analysis of a blend containing two flavonoids, namely luteolin and rutin, along with quercetin, all in a molar ratio of 1:1. The spectra for the mixture of luteolin and quercetin are depicted in pink, while the blend of rutin and quercetin appears in cyan. Additionally, the spectra for the individual components—luteolin (blue), rutin (yellow), and quercetin (green)—were sourced from the results in Figure 4, with each analyte measured at a concentration of 10^−3^ M. (**b**) The SERS spectra represent the multiplex analysis of a combination of four flavonoids—luteolin, rutin, quercetin, and kaempferol—all in a molar ratio of 1:1:1:1.

To validate the feasibility of TiO_2_@AgNPs for differentiating flavonoids present in *AS* extracts, we carried out a proof-of-concept experiment using standard luteolin, quercetin, kaempferol, and rutin, as described in Section 3.2. We primarily distinguish overlapping SERS signals by identifying the unique characteristic peaks of each flavonoid. Initially, the SERS spectra of the bi-analyte mixtures of luteolin or rutin with quercetin, both at a 1:1 molar ratio, were obtained using TiO_2_@AgNPs simultaneously. As depicted in Figure 6a, the individual SERS spectra of these three flavonoids, extracted from Figure 4 at an equivalent concentration (10^−3^ M), served as a benchmark for dual analysis. We detected dual SERS peaks corresponding to the flavonoids. In the mixture of luteolin and quercetin, the peaks at 1575 cm^−1^, 741 cm^−1^, 684 cm^−1^, and 424 cm^−1^ were associated with the C=C deformation modes of luteolin, while the peaks at 1504 cm^−1^, 1258 cm^−1^, and 1222 cm^−1^ were associated with the OH in-plane bending modes of luteolin. Conversely, the peaks at 935 cm^−1^, 845 cm^−1^, and 486 cm^−1^ were assigned to quercetin. In contrast, the mixture of rutin and quercetin exhibited the characteristic peaks for rutin at 1652 cm^−1^, 1500 cm^−1^, 878 cm^−1^, and 424 cm^−1^, and for quercetin, it exhibited peaks at 1173 cm^−1^, 1110 cm^−1^, and 642 cm^−1^. Notably, although both mixtures contained the same proportion of quercetin, the vibration patterns of the characteristic peaks detected differed significantly. Moreover, the multiplex analysis of the aforementioned four flavonoids in a 1:1:1:1 molar ratio was further investigated using the TiO_2_@AgNPs. Compared with the single-analyte SERS spectra extracted from Figure 4, the specific peaks of each component were also effectively distinguished, indicating that this method can accurately identify multiple flavonoids in mixed systems (Figure 6b). This finding further validates the capability of the proposed TiO_2_@AgNPs for *AS* analysis. However, this study did not perform specialized statistical analysis on quantitative accuracy, which will be addressed in future work to further validate the accuracy of the method.

### 3.4. SERS Discrimination of AS from Different Origins

**Figure 7:**
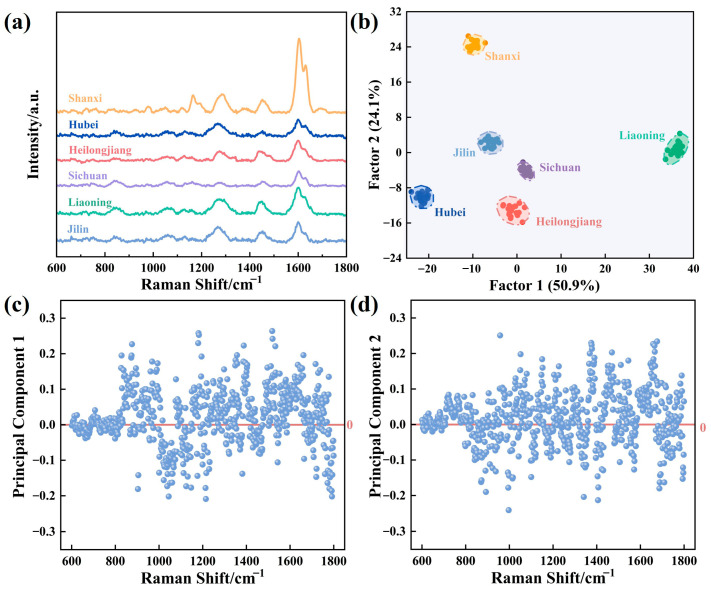
(**a**) SERS spectra of *AS* root extracts from six origins: Shanxi, Hubei, Heilongjiang, Sichuan, Liaoning, and Jilin. (**b**) PCA diagram of the SERS data for the specific identification of the six origins. The loading plot of principal component 1 (**c**) and component 2 (**d**) from PCA of the specific SERS identification of *AS*.

To evaluate the efficacy of TiO_2_@AgNPs in distinguishing regional variations in *AS*, we conducted SERS measurements on extracts collected from six geographically distinct regions in China: Sichuan, Shanxi, Liaoning, Jilin, Hubei, and Heilongjiang. A comprehensive analysis of the SERS spectra from these regions revealed that the samples exhibited highly similar spectral profiles in their peaks (Figure 7a). Notably, the optimal SERS results were obtained from samples originating in Shanxi province. In contrast, the SERS spectra from the other five areas demonstrated significant spectral overlap, which may be attributed to signal interference caused by polysaccharides, organic acids, and other bioactive compounds present in *AS*. These compounds could obscure or attenuate specific peaks in the root extract spectra. The SERS spectra of the extracts reveal vibrational signatures consistent with key flavonoid constituents. A peak at 1604 cm^−1^ corresponds to in-plane deformations of the ring, OH bending, and C=O stretching modes observed in luteolin, rutin, kaempferol, and quercetin, while the feature at 1285 cm^−1^ relates to CH in-plane bending vibrations of luteolin and rutin. Additional assignments include a COH in-plane bending mode of quercetin at 1165 cm^−1^ and a CH out-of-plane bending vibration of kaempferol at 980 cm^−1^. Moreover, the extracts from each origin were tested 18 times, and the SERS spectra along with the resulting histograms related to the corresponding relative standard deviation (RSD) are presented in Figures S6–S11, demonstrating better SERS signal repeatability.

To further extract the SERS spectral features for accurate origin recognition, we employed principal component analysis (PCA) to investigate the effects of different provinces based on the intensities rather than the positions of characteristic SERS peaks. The differences in the intensity of these characteristic peaks reflect changes in the relative concentrations of flavonoids, thereby providing a basis for regional differentiation. As illustrated in Figure 7b, *AS* from six origins could be effectively classified using PCA, with principal component 1 explaining 50.9% of the total variance, thereby confirming a relatively good separation of the origins due to differences in flavonoid concentrations. Additionally, the loading plots for principal components 1 and 2 are presented in Figure 7b,c, where the horizontal axis represents the variables and the vertical axis denotes the discriminant coefficients of these variables for principal components 1 and 2. However, the values of all discriminant coefficients remain below 0.3 within the spectral range of 600–1800 cm^−1^, indicating that no prominent Raman peak dominates the descriptors of factors 1 and 2. It is important to note that other bioactive compounds, such as polysaccharides and organic acids present in *AS* extracts, may introduce signal interference, resulting in overlapping SERS spectra among samples from certain regions. Nevertheless, this did not impede the effective classification of different origins by PCA, further corroborating the conclusion that differences in flavonoid concentrations are the main contributors to signal changes.

## 4. Conclusions

In this work, we designed and fabricated TiO_2_@AgNP nanocomposites as a novel SERS sensor for the detection of bioactive components, specifically flavonoids rich in hydroxyl groups, which serve as quality indicators in *Acanthopanax senticosus*. The TiO_2_ core provides abundant sites for molecular adsorption, while the surrounding AgNPs create ‘hot spots’ for SERS enhancement. The TiO_2_@AgNPs demonstrate effective recognition of four flavonoids and exhibit excellent signal repeatability, highlighting their impressive SERS capabilities for quantitative assessments. Specifically, the minimal concentrations of detection were found to be 10^−6^ M for luteolin and rutin, 10^−5^ M for kaempferol, and 5 × 10^−6^ M for quercetin. Moreover, this SERS sensor supports dual or multiplex analysis of these flavonoids, allowing for the preliminary identification of *Acanthopanax senticosus* extracts based on significant flavonoid components. As part of a proof of concept for practical uses, the TiO_2_@AgNP sensor successfully distinguished *AS* from six geographically diverse regions across China. The preparation of TiO_2_@AgNPs involves a multi-step chemical synthesis process, which increases complexity and cost in large-scale production, potentially hindering its adoption in laboratories or production environments with limited resources. However, compared to traditional methods such as chromatography and mass spectrometry, this SERS platform offers potential advantages in terms of analysis speed and ease of operation. Therefore, this established methodology positions the TiO_2_@AgNP-sensing platform for future applications in categorizing and differentiating traditional Chinese medicine, as well as for assessing quality and medicinal worth.

## Data Availability

Data is contained within the article.

## Supplementary Materials

The following supporting information can be downloaded at: https://www.mdpi.com/article/10.3390/bios15080536/s1, Figure S1: Solid UV-Vis absorption spectra of pure TiO_2_ and TiO_2_@AgNPs with different molar ratios of TiO_2_ to Ag; Figure S2: SERS spectra for luteolin (10^−4^ mol/L) obtained from AgNPs, TiO_2_, and TiO_2_@AgNPs with molar ratios of TiO_2_ to Ag 2 × 10^1^, respectively; Figure S3: SEM images (a,c,e,g,i) and size distribution of TiO_2_@AgNPs (b,d,f,h,j) with molar ratios of TiO_2_ to Ag 2 (a,b), 2 × 10^2^ (c,d), 2 × 10^3^ (e,f), 2 × 10^4^ (g,h), and pure TiO_2_ (i,j); Figure S4: Theoretical simulation of the electromagnetic field enhancement distribution of (a) TiO_2_ and (b) TiO_2_@AgNPs with molar ratios of TiO_2_ to Ag 2 × 10^1^; Figure S5: EDS mapping images of TiO_2_@AgNPs with molar ratios of TiO_2_ to Ag 2 × 10^1^; Figure S6: (a) SERS spectra of 18 samples randomly collected from *AS* extracts derived from Shanxi. (b) Three-dimensional bar histograms illustrating the intensity distributions for 1452 cm^−1^ (purple), 1280 cm^−1^ (grey), 1630 cm^−1^ (pink), and 1604 cm^−1^ (blue), along with the corresponding RSDs calculated from (a); Figure S7: (a) SERS spectra of 18 samples randomly collected from *AS* extracts derived from Hubei. (b) Three-dimensional bar histograms illustrating the intensity distributions for 1452 cm^−1^ (purple), 1280 cm^−1^ (grey), 1630 cm^−1^ (pink), and 1604 cm^−1^ (blue), along with the corresponding RSDs calculated from (a); Figure S8: (a) SERS spectra of 18 samples randomly collected from *AS* extracts derived from Heilongjiang. (b) Three-dimensional bar histograms illustrating the intensity distributions for 1452 cm^−1^ (purple), 1280 cm^−1^ (grey), 1630 cm^−1^ (pink), and 1604 cm^−1^ (blue), along with the corresponding RSDs calculated from (a); Figure S9: (a) SERS spectra of 18 samples randomly collected from *AS* extracts derived from Sichuan. (b) Three-dimensional bar histograms illustrating the intensity distributions for 1452 cm^−1^ (purple), 1280 cm^−1^ (grey), 1630 cm^−1^ (pink), and 1604 cm^−1^ (blue), along with the corresponding RSDs calculated from (a); Figure S10: (a) SERS spectra of 18 samples randomly collected from *AS* extracts derived from Liaoning. (b) Three-dimensional bar histograms illustrating the intensity distributions for 1452 cm^−1^ (purple), 1280 cm^−1^ (grey), 1630 cm^−1^ (pink), and 1604 cm^−1^ (blue), along with the corresponding RSDs calculated from (a); Figure S11: (a) SERS spectra of 18 samples randomly collected from *AS* extracts derived from Jilin. (b) Three-dimensional bar histograms illustrating the intensity distributions for 1452 cm^−1^ (purple), 1280 cm^−1^ (grey), 1630 cm^−1^ (pink), and 1604 cm^−1^ (blue), along with the corresponding RSDs calculated from (a); Table S1: The assignments of SERS peaks for luteolin in this work [37]; Table S2: The assignments of SERS peaks for quercetin in this work [40,41]; Table S3: The assignments of SERS peaks for kaempferol in this work [41,42]; Table S4: The assignments of SERS peaks for rutin in this work [43,44].
