# Supplementary material for: Bifunctional TiO2@AgNP Superstructures as a SERS-Sensing Platform for Identifying Flavonoids in Chinese Herbal Medicine
Source: Biosensors (Basel). 2025 Aug 15;15(8):536. doi: 10.3390/bios15080536 (PMC12384181; doi:10.3390/bios15080536)
Supplement: Supplementary file 1 [file biosensors-15-00536-s001.zip › biosensors-3777283-supplementary.pdf]

biosensors

Supplementary Materials

Bifunctional TiO_2@AgNP Superstructures as a SERS-Sensing Platform for Identifying Flavonoids in Chinese Herbal Medicine

Yulin Li [1], Junbo Li [1,*], Haisu Wang [1], Shaorui Qi [1], Zhehao Zhang [1], Yaqiu Wang [1], Ying Wang [1] and Wei Ji [1,2,*]

1 College of Chemistry, Chemical Engineering and Resource Utilization, Northeast Forestry University, Harbin 150040, China; liyulin@nefu.edu.cn (Y.L.); haisuwang@nefu.edu.cn (H.W.); 2023110889@nefu.edu.cn (S.Q.); easymony_zhang@nefu.edu.cn (Z.Z.); laurawang@nefu.edu.cn (Yaqiu Wang); wangying@nefu.edu.cn (Ying Wang)
2 School of Chemical Engineering, Dalian University of Technology, Dalian 116024, China
* Correspondence: lijunbo91@nefu.edu.cn (J.L.); jiwei@dlut.edu.cn (W.J.)

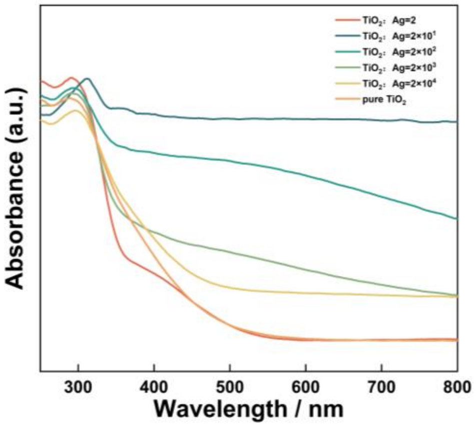

Figure S1. Solid UV-Vis absorption spectra of pure TiO_2 and TiO_2@AgNPs with different molar ratios of TiO_2 to Ag.

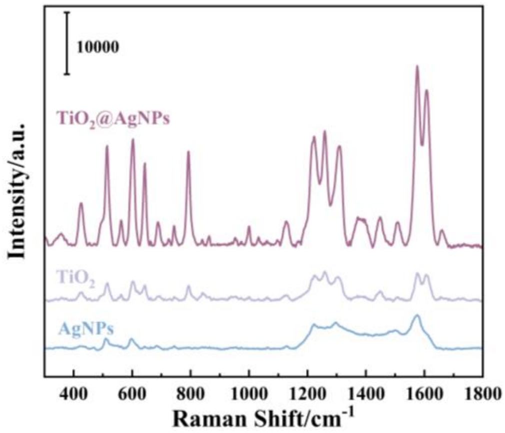

Figure S2. SERS spectra for luteolin (10^{-4} mol/L) obtained from AgNPs, TiO_2, and TiO_2@AgNPs with molar ratios of TiO_2 to Ag 2×10^{1}, respectively.

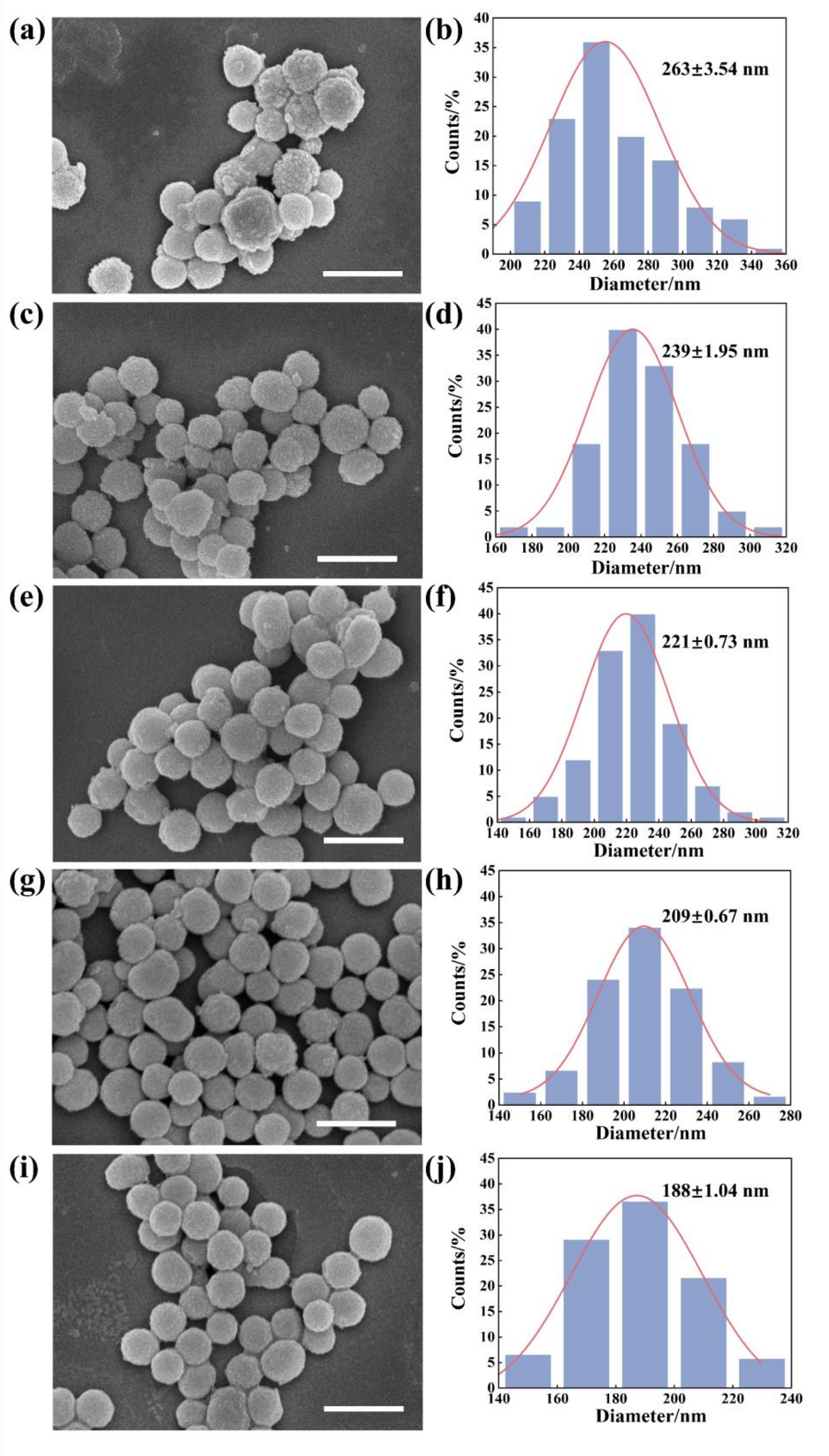

Figure S3. SEM images (a, c, e, g, i) and size distribution of TiO_2@AgNPs (b, d, f, h, j) with molar ratios of TiO_2 to Ag 2 (a, b), 2×10^2 (c, d), 2×10^3 (e, f), 2×10^4 (g, h), and pure TiO_2 (i, j). Scale bars: 500 nm.

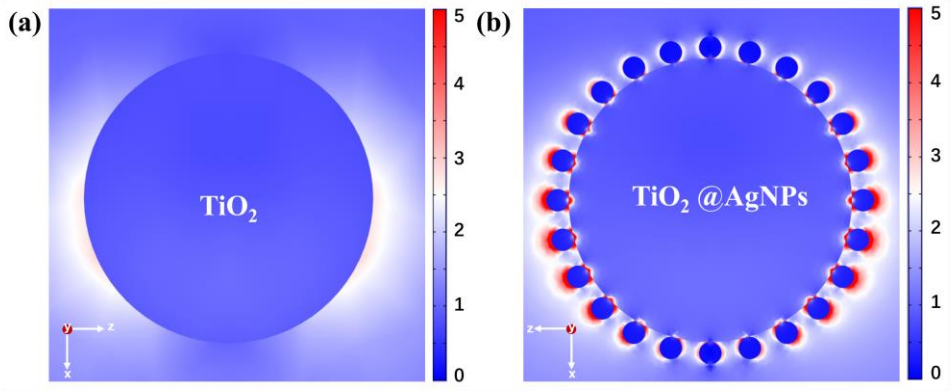

Figure S4. Theoretical simulation of the electromagnetic field enhancement distribution of (a) TiO_2 and (b) TiO_2@AgNPs with molar ratios of TiO_2 to Ag 2×10^1.

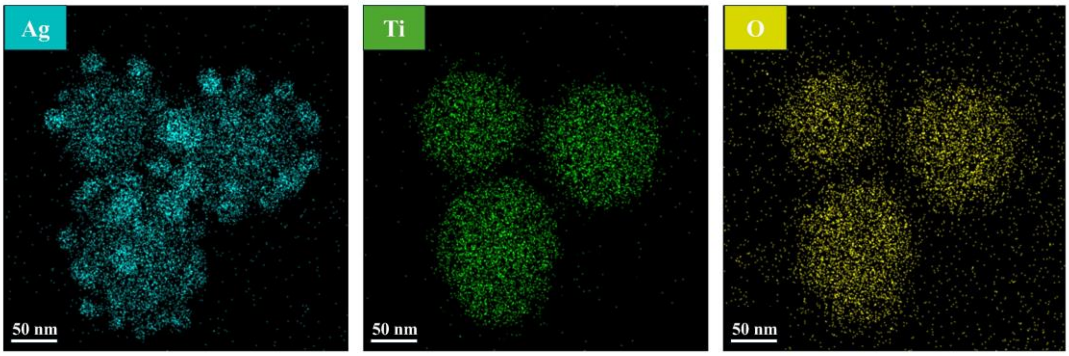

Figure S5. EDS mapping images of TiO_2@AgNPs with molar ratios of TiO_2 to Ag 2×10^1.

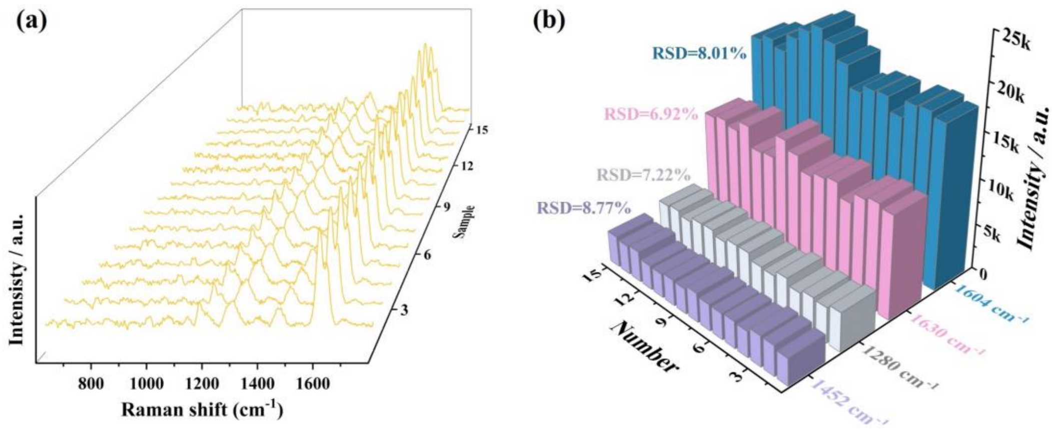

Figure S6. (a) SERS spectra of 18 samples randomly collected from *AS* extracts derived from Shanxi. (b) Three-dimensional bar histograms illustrating the intensity distributions for 1452 cm^{-1} (purple), 1280 cm^{-1} (grey), 1630 cm^{-1} (pink), and 1604 cm^{-1} (blue), along with the corresponding RSDs calculated from (a).

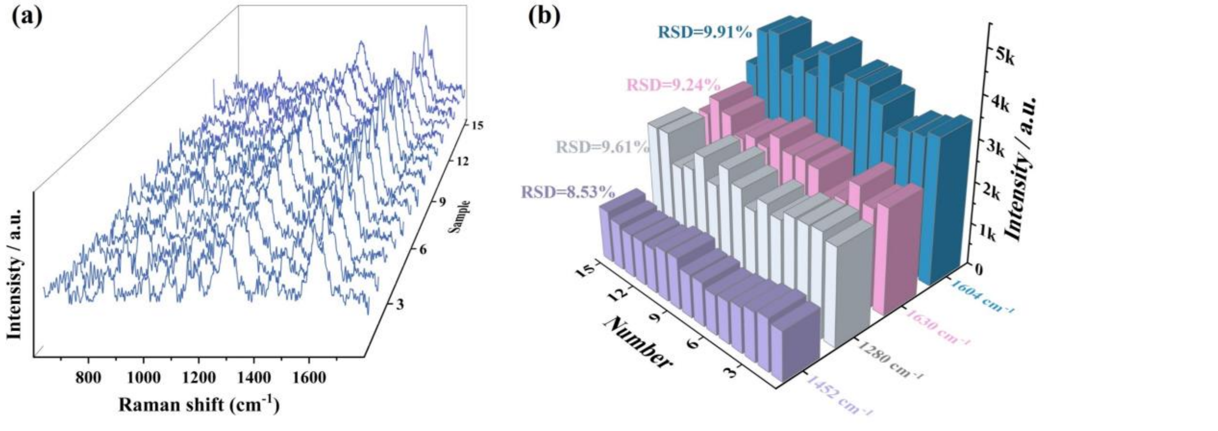

Figure S7. (a) SERS spectra of 18 samples randomly collected from *AS* extracts derived from Hubei. (b) Three-dimensional bar histograms illustrating the intensity distributions for 1452 cm^{-1} (purple), 1280 cm^{-1} (grey), 1630 cm^{-1} (pink), and 1604 cm^{-1} (blue), along with the corresponding RSDs calculated from (a).

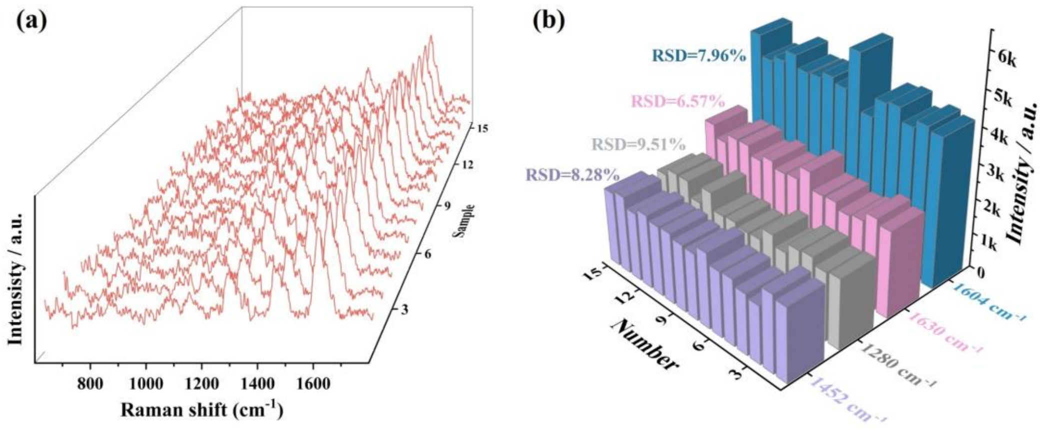

Figure S8. (a) SERS spectra of 18 samples randomly collected from *AS* extracts derived from Heilongjiang. (b) Three-dimensional bar histograms illustrating the intensity distributions for 1452 cm^{-1} (purple), 1280 cm^{-1} (grey), 1630 cm^{-1} (pink), and 1604 cm^{-1} (blue), along with the corresponding RSDs calculated from (a).

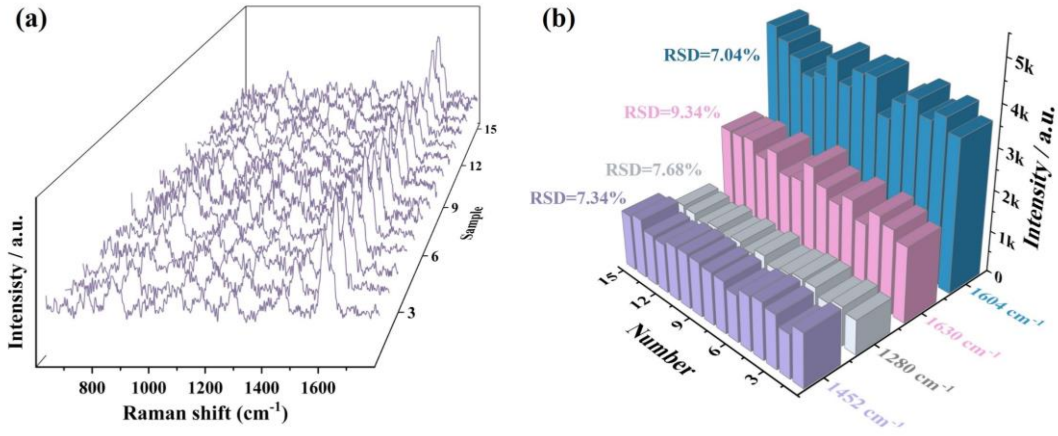

Figure S9. (a) SERS spectra of 18 samples randomly collected from *AS* extracts derived from Sichuan. (b) Three-dimensional bar histograms illustrating the intensity distributions for 1452 cm^{-1} (purple), 1280 cm^{-1} (grey), 1630 cm^{-1} (pink), and 1604 cm^{-1} (blue), along with the corresponding RSDs calculated from (a).

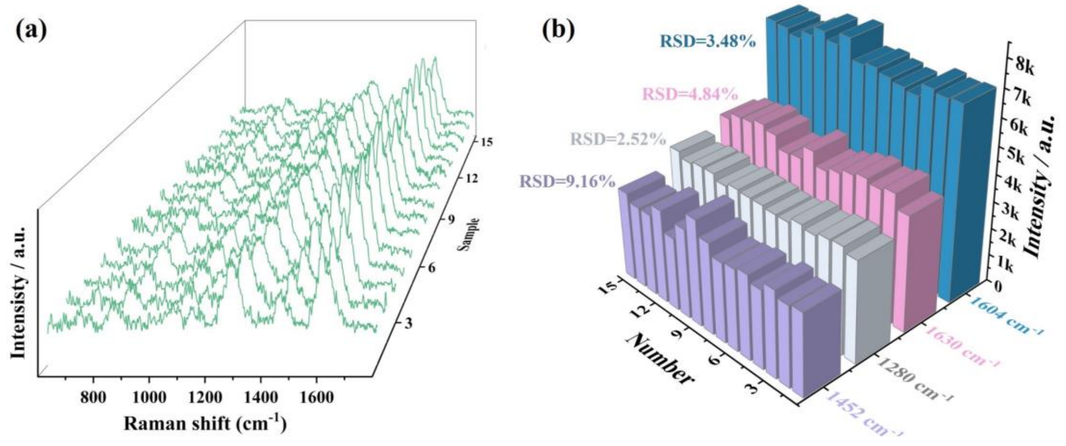

Figure S10. (a) SERS spectra of 18 samples randomly collected from *AS* extracts derived from Liaoning. (b) Three-dimensional bar histograms illustrating the intensity distributions for 1452 cm^{-1} (purple), 1280 cm^{-1} (grey), 1630 cm^{-1} (pink), and 1604 cm^{-1} (blue), along with the corresponding RSDs calculated from (a).

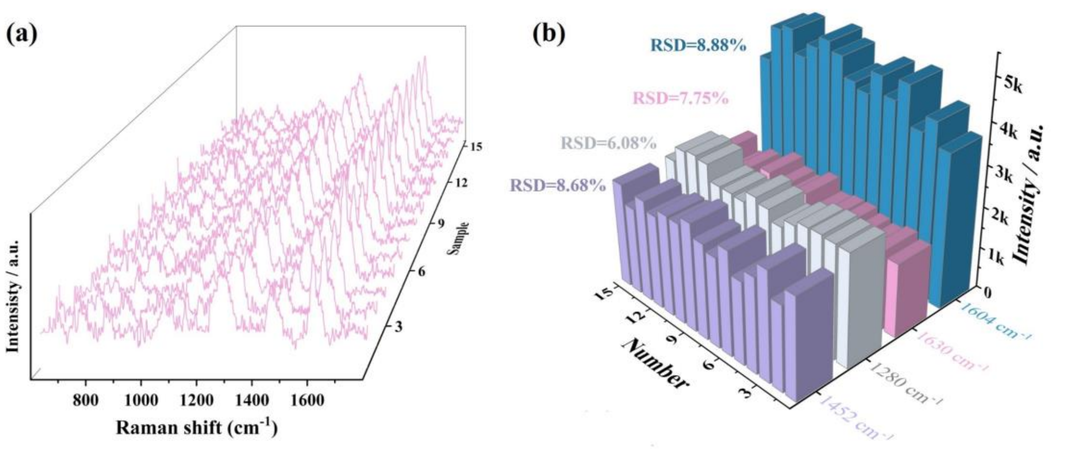

Figure S11. (a) SERS spectra of 18 samples randomly collected from *AS* extracts derived from Jilin. (b) Three-dimensional bar histograms illustrating the intensity distributions for 1452 cm^{-1} (purple), 1280 cm^{-1} (grey), 1630 cm^{-1} (pink), and 1604 cm^{-1} (blue), along with the corresponding RSDs calculated from (a).

Table S1. The assignments of SERS peaks for luteolin in this work.

Analyte	Peaks in reference (cm^{-1})	Peaks in this work (cm^{-1})	Assignment	References
Luteolin	424	424	C=C in-plane deformations of ring	[37]
	518	512	C6H, 7OH in-plane bending	
	566	562	3OH, 7OH in-plane bending	
	607	602	Ring deformations	
	645	641	C=C in-plane deformations of ring	
	686	687	C=C in-plane deformations of ring	
	734	741	C=C deformations of ring	
	798	791	Ring breath	
	1002	997	C6H, C8H, C2'H, C5'H in-plane bending	
	1131	1124	C3H, C5'H, C6'H, 4'OH in-plane bending	
	1224	1222	C5'H, 4'OH in-plane bending	
	1270	1258	C3H, 5OH in-plane bending	
	1304	1302	Ring breath	
	1447	1446	5OH in-plane bending	
	1504	1504	3'OH in-plane bending	
	1576	1575	C2=C3, ring stretching	
	1612	1606	C=O stretching; 5OH bending	
	1660	1654	C2=C3, C=O stretching; 5OH bending	

Table S2. The assignments of SERS peaks for quercetin in this work.

Analyte	Peaks in reference (cm^{-1})	Peaks in this work (cm^{-1})	Assignment	References
Quercetin	521	521	C=C in-plane deformations of ring	[40,41]
	608	601	C=C in-plane deformations of ring	
	662	642	CC out-of-plane deformations of ring, CH bending	
	711	723	C=C in-plane deformations of ring	
	845	845	C=C in-plane stretching of ring	
	1002	1002	CH in-plane bending, C-O-C bending	
	1115	1110	3OH, 3'OH,4'OH, CH bending	
	1186	1173	C7OH, C8H in-plane bending	
	1319	1320	3OH, 5OH, 3'OH in-plane bending	
	1355	1365	4'OH bending, CH in-plane bending	
	1433	1442	3OH, 5OH, 7OH bending	
	1598	1607	C=O stretching, OH in-plane bending, ring breath	

Table S3. The assignments of SERS peaks for kaempferol in this work.

Analyte	Peaks in reference (cm^{-1})	Peaks in this work (cm^{-1})	Assignment	References
Kaempferol	519	519	C=C in-plane deformations of ring	[41,42]
	586	583	C-C stretching of ring	
	642	642	CC out-of-plane deformations of ring, CH bending	
	977	977	CH out-of-plane bending of ring	
	1172	1187	C7OH and C8H in-plane bending	
	1369	1371	3,5OH in-plane bending	
	1450	1443	3,5OH, CH ip bend	
	1561	1605	in-plane deformations of ring, C=O stretching, 5OH bending	

Table S4. The assignments of SERS peaks for rutin in this work.

Analyte	Peaks in reference (cm^{-1})	Peaks in this work (cm^{-1})	Assignment	References
Rutin	574	598	Deformations of ring	[43,44]
	658	658	Ring breath	
	793	792	CH wagging at ring, ring breathing	
	850	849	C=C stretching	
	947	938	C=C stretching	
	998	996	C=C=C bending, CC stretching	
	1130	1130	C=O stretching	
	1299	1297	COH bending, CCH bending, CH rocking	
	1365	1362	C=O, C=C stretching, ring breathing,	
	1502	1500	C=C stretching	
	1557	1562	C=C stretching	
	1608	1605	C=O stretching	
	1658	1652	C=O stretching	
